# Effectiveness of Sterilized Symbiotic Drink Containing *Lactobacillus helveticus* Comparable to Probiotic Alone in Patients with Constipation-Predominant Irritable Bowel Syndrome

**DOI:** 10.1007/s10620-019-05695-3

**Published:** 2019-06-17

**Authors:** Mohd Fyzal Bahrudin, Rafiz Abdul Rani, Azmi Mohd Tamil, Norfilza Mohd Mokhtar, Raja Affendi Raja Ali

**Affiliations:** 1 Gastroenterology Unit, Department of Medicine, Faculty of Medicine, Universiti Kebangsaan Malaysia, Jalan Yaacob Latiff, Bandar Tun Razak, 56000 Cheras, Kuala Lumpur Malaysia; 2 Gastroenterology Unit, Faculty of Medicine, Universiti Teknologi MARA, 47000 Sungai Buloh, Selangor Malaysia; 3 Department of Community Health, Faculty of Medicine, Universiti Kebangsaan Malaysia, Jalan Yaacob Latif, Bandar Tun Razak, 56000 Cheras, Kuala Lumpur Malaysia; 4 Department of Physiology, Faculty of Medicine, Universiti Kebangsaan Malaysia, Jalan Yaacob Latif, Bandar Tun Razak, 56000 Cheras, Kuala Lumpur Malaysia; 5 GUT Research Group, Faculty of Medicine, Universiti Kebangsaan Malaysia, Cheras, Kuala Lumpur Malaysia

**Keywords:** Irritable bowel syndrome, Constipation, Probiotics, *Lactobacillus helveticus*, Polydextrose

## Abstract

**Background:**

This study aimed to objectively investigate whether the addition of polydextrose to sterilized probiotic containing *Lactobacillus helveticus* will confer benefits to constipation-predominant irritable bowel syndrome patients.

**Methods:**

A total of 163 patients were randomized into two groups: Group A to consume 350 mL of sterilized probiotic with 5.85 g polydextrose daily for 1 week and Group B without polydextrose. Intestinal transit time, fecal pH, fecal weight, and modified *Garrigues* questionnaires for pre- and post-consumption were assessed.

**Results:**

Median intestinal transit time was significantly reduced from 58 (IQR 43–72) to 45 (IQR 24–59) hours and 48 (IQR 31–72) to 30 (IQR 24–49) hours for Groups A and B, respectively (*p *< 0.01). Fecal pH for Groups A and B was significantly reduced from 6.57 ± 0.96 to 6.13 ± 0.95 (*p* = 0.003) and 6.58 ± 1.0 to 5.87 ± 0.83 (*p* < 0.001), respectively. Fecal weight for Group A was significantly increased from 8 g ± 6.4 g to 9.8 g ± 7.6 g (*p* = 0.003), but it was reduced for Group B from 13.3 g ± 19.4 g to 11.2 g ± 6.6 g (*p* = 0.308). Constipation-related symptoms were significantly improved for both groups.

**Conclusions:**

The addition of polydextrose to sterilized probiotic containing *L. helveticus* did not show significant benefits to constipation-predominant irritable bowel syndrome patients. However, daily consumption of sterilized probiotic containing *L. helveticus* with or without polydextrose for a week alleviated constipation-related symptoms and objectively reduced both fecal pH and intestinal transit time.

## Introduction

Irritable bowel syndrome (IBS) is a major functional gastrointestinal disorder seen in daily clinical practice. Diagnostic criteria for IBS had continuously evolved with the first formal criteria by Manning et al. in 1978 to the latest Rome IV criteria published in June 2016 [1, 2]. Generally, the prevalence of IBS is at 10–20% and a meta-analysis conducted in 2012 utilizing the diverse diagnostic criteria from Manning to Rome III criteria had reported the global prevalence to be approximately 11.2% [3]. In multi-ethnic Malaysia populations, the prevalence is at 10.9–15.8% [4–6].

A person is diagnosed to have IBS if he or she has recurrent abdominal pain or discomfort for at least 3 days per month in the past 3 months. This symptom has to be associated with at least two of the following: relief with defecation, onset of symptoms associated with altered fecal frequency, or altered fecal form. Onset of symptoms has to be more than 6 months prior to diagnosis [7]. Constipation-predominant IBS (IBS-C) is defined as “IBS with the passing of hard stools more than 25% of the time and loose stools less than 25% of the time” [8]. The risk factors identified for IBS-C include younger age, increased psychological stress, incorrect diet, and sedentary lifestyles [9, 10]. Besides that, multiple causes were implicated in the pathophysiology of IBS, including disorder of gut–brain interaction, visceral hypersensitivity, serotonin dysregulation, microscopic inflammation, and gut dysbiosis [8, 11–13].

It has been demonstrated that the diversity of microbial populations is reduced, particularly the populations of *Lactobacilli* sp. and *Bifidobacteria* sp. [14]. Probiotics is defined as “live microorganisms that grant health benefits to the host if given in the right doses” by the Food and Agriculture Organization of the United Nations and The World Health Organization [15]. Cultured milk drink is a form of food product produced by bacteria fermentation of milk. It was reported to aid digestive movements, increase defecation frequency, reduce abdominal pain or discomfort, and reduce flatulence [16, 17]. Prebiotic is a substrate that selectively uses a host microorganism to produce a health benefit. Examples of prebiotics are polydextrose and insulin. The addition of fiber may change the gut microbiota constitution, such as increase in the *Lactobacillus* populations [18, 19]. Polydextrose is a water-soluble glucose polymer partially fermented in the large intestine and is resistant to digestion by human intestine. By incorporating polydextrose, it is postulated to promote a shorter intestinal transit time, improve stool consistency, soften the feces, and improve the ease of defecation with no adverse effects. Combination of probiotic and prebiotic is known as symbiotic.

Previous study by Rees et al. reported that increasing intake of non-starch polysaccharide significantly increases mean stool wet weight among IBS patients, whereas Magro et al. reported a product containing yogurt with polydextrose, *Bifidobacterium lactis* HN019 and *Lactobacillus acidophilus* NCFM^®^, had significantly shortened intestinal transit time after 2 weeks of intervention [20, 21]. To date, there is still a lack of studies comparing the effects of sterilized probiotic with and without added prebiotic among IBS-C patients in the form of clinical symptoms and biochemical changes to the feces [22]. Hence, this study was embarked to objectively assess this option as a viable and feasible therapeutic option for IBS-C patients.

## Materials and Methods

### Subjects

This prospective, double-blind randomized controlled trial was conducted from January 2016 to July 2017. Patients fulfilling the Rome III IBS-C criteria were recruited from the Gastroenterology clinic and Endoscopy Unit at Universiti Kebangsaan Malaysia Medical Centre, Kuala Lumpur, Malaysia. Sample size calculation was performed according to a study by Min et al. on assessing IBS-C symptoms improvement [23]. Sixty-three pairs of subjects were required to be studied to be able to reject the null hypothesis that this response difference is zero with probability (power) of 0.8. The type I error probability associated with this test of this null hypothesis is 0.05. Therefore, a total of 160 subjects were recruited for this study with equal number on both arms (80 subjects) to be able to reject the null hypothesis for both designs of the study. The sample size was calculated based on Power and Sample Size (PS3) (version 3.1.2, Nashville, USA) [16, 24].

The exclusion criteria included age less than 18 years old, constipation due to other medical illnesses such as diabetes mellitus, inflammatory bowel disease, hypothyroidism, colorectal carcinoma, neurological diseases, major depression, pregnancy, breastfeeding women, and patients who were on chronic opioids or anti-depressants. Subjects must not have consumed any antibiotics, probiotics, prebiotics, symbiotics, and/or laxatives less than 2 weeks before recruitment. Lactose-intolerant patients were also excluded from this clinical trial through identification by clinical assessments during IBS screening. Our center did not perform hydrogen breath test to exclude lactose intolerance. Lactose intolerance is suggested when there is a resolution of symptoms with the elimination of lactose-containing food products and resumption of symptoms when lactose-containing food products reintroduction [25, 26].

**Fig. 1:**
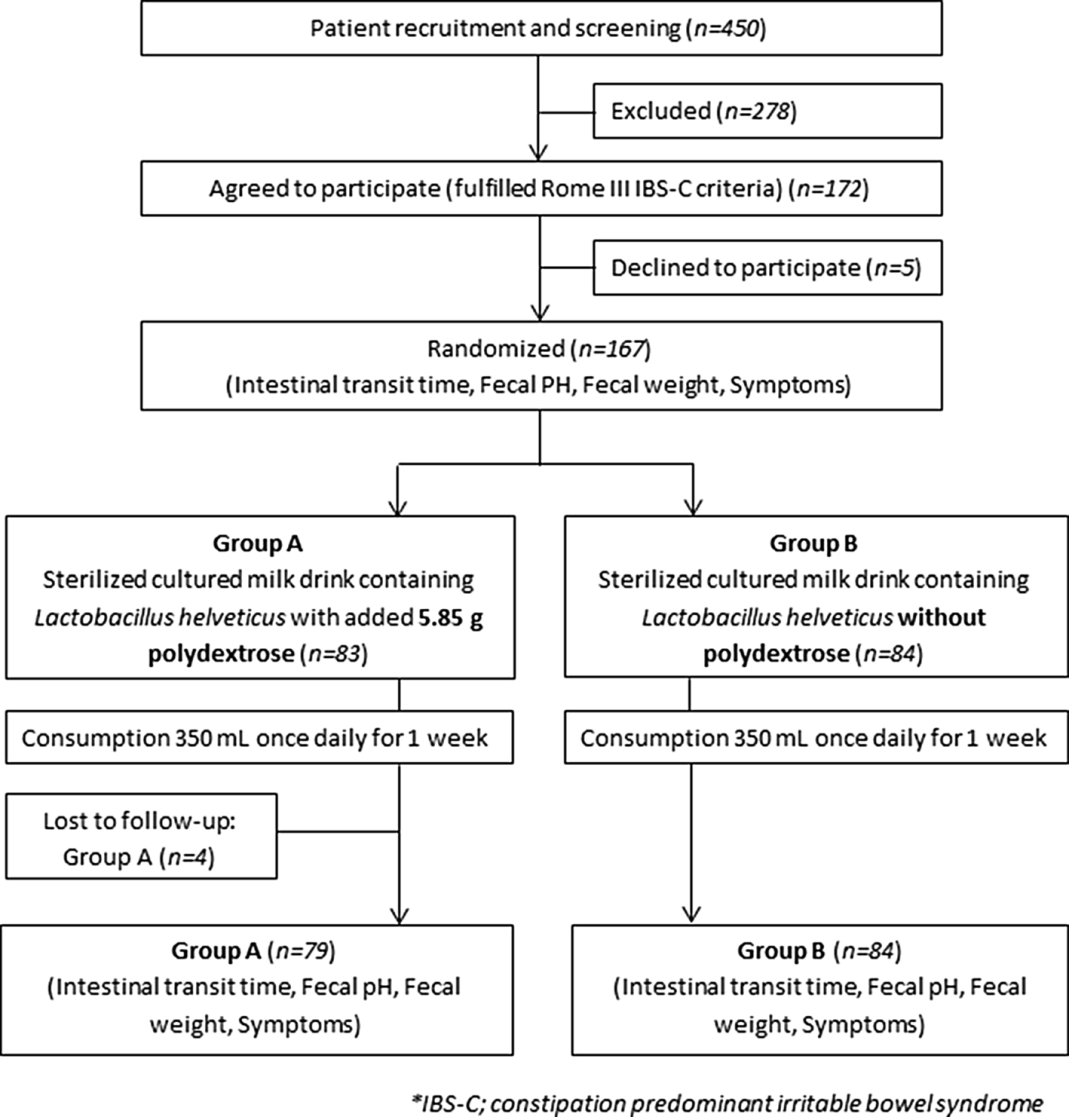
Flowchart of study

Simple randomization was performed using a random number table. The subjects were randomized into two groups: Group A or symbiotic group, which received 350 mL of daily sterilized probiotic with *L. helveticus* and added 5.85 g polydextrose, and Group B or probiotic group, which received 350 mL of daily sterilized probiotic with *L. helveticus* without polydextrose for a total of 7 days (Fig. 1). During the period of study, subjects were advised not to take any laxatives, prebiotic supplement, yogurt, or other probiotic during the 1-week study period.

At the point of pre-consumption and upon completion of 7-day consumption, the symptoms and intestinal transit time were assessed with the collected stool samples. The subjects were reminded by phone to ensure compliance and empty bottles of probiotic were returned after completion.

### Symbiotic and Probiotic Drink

Test products were packaged, labeled, and blinded to investigators and subjects. Simple randomization was performed to allocate subjects into two separate groups: Group A and Group B. For each bottle of 350 mL of sterilized probiotic, it contained *L. helveticus* with or without added 1.5 g/100 mL of polydextrose. A 0.5 g extra of polydextrose is added by taking into consideration the degradation compensation and preparation loss. The amount of *L. helveticus* is minimal as all probiotics were sterilized. Seven bottles were given to each subject. For both products, they were identical in weight, color, smell, taste, and package. These are the ingredient list: water, sugar, skimmed milk powder (cow), stabilizers (polydextrose (ins 1200 and ins 440), fermented milk [water, acidity regulator (ins 330), skimmed milk powder (cow), and lactobacillus], acidity regulators (ins 270 and ins 331 (iii)), soybean fiber, and flavoring. One serving contained 350 mL of cultured drink. For each 350 mL, there are 203 kcal/853 kJ energy, 45.5 g carbohydrate, 36.8 g total sugar, 36.8 g sucrose, 5.3 g lactose, 3.5 g protein, 0.0 g fat, 5.8 g dietary fiber, and 69 mg sodium. All doctors, research staff, and subjects involved were unaware of the treatment administered to the subject.

### Protocol to Determine Intestinal Transit Time (ITT)

Red carmine capsule, a non-toxic food colorant, was used to determine the ITT [27]. Following consumption, there was a red discoloration of the stool. The ITT was determined from the time of ingestion to the first stool discoloration. Upon completion of 7 days of the test products, the subjects were required to repeat the ITT test.

### Determination of Fecal Weight and Fecal pH

Stool samples were collected twice; once prior to consumption and subsequent collection upon first defecation after consumption of the last treatment drink. Each subject was asked to fill 20 mL of fresh stool sample in a standardized stool container. The changes in fecal weight in both groups after the intervention were calculated, and the significance of the changes in fecal weight between the groups was analyzed. The volume of 20 mL was standardized and preferred due to the difficulty in collecting the total stool for the whole day and logistic reasons. Fecal weight was measured in gram (g), while pH was measured utilizing litmus pH paper.

### Constipation-Related Symptoms Assessment

To objectively assess constipation-related symptoms, we used *Garrigues* constipation questionnaires [28]. There are 21 components in the questionnaires, which include passing of hard stool, straining during defecation, incomplete emptying sensation feeling after bowel movement, blockage feeling in the anus, the need of pressing around perineum to assist defecation, spending more than 10 min in the toilet to defecate, and the number of bowel movements per week. All questions were graded as never, sometimes (less than 25% of time), often (more than 25% of time), or always.

### Statistical Analysis

Analysis was performed using IBM SPSS Statistics version 23 (IBM Corp., New York, USA), and *p *< 0.05 was considered to be statistically significant. For fecal pH and fecal weight, paired *t* test was used pre- and post-intervention separately based on intervention groups. General linear model with repeated measures was used for the statistical analyses pre- and post-intervention between the different groups for fecal pH and fecal weight. The fecal ITT was not normally distributed; therefore, Wilcoxon rank-sum test was used separately according to intervention group. Mann–Whitney test was also used for statistical analysis between different intervention groups for ITT assessment.

## Results

### Demographics and Socioeconomic

Initially, a total of 450 subjects were evaluated from clinic list. A total of 172 subjects fulfilled the diagnostic criteria for IBS-C. Out of 172 subjects, five declined participation while four subjects defaulted follow-up. A total of 163 subjects completed the study with 79 in Group A (symbiotic) and the remaining 84 subjects in Group B (probiotic). There were four subjects who withdrew from the study; one subject was due to intake of a different probiotic, and three of the subjects were lost to follow-up. These results are presented in Fig. 1.

The majority of IBS-C subjects (78.6%) were women then followed by men (21.4%). Three major ethnicities were recruited: Malays (73.1%), Chinese (24.5%), and Indians (1.2%). The median age for Group A was 34 years old, whereas for Group B was 27 years old. Median age was used as the subjects were not equally distributed. Based on the educational levels of the subjects, a higher percentage (74.9%) received tertiary level of education. However, 45.4% had low income of less than ringgit Malaysia (RM) 1500 per month and 54.6% had income more than RM 1500 per month with no correlation with their respective education level. In terms of social status, 96.3% were non-smokers and 99.4% never consumed alcohol. Dietary history among the recruited subjects showed 62% of them consumed low-fiber diet, 35% had moderate fiber intake, and only 3.1% had high-fiber intake. Pertaining to their lifestyles, 54% claimed that they sometimes had physical activity in their daily lives, 12.3% habitually performed exercise, and 33.7% did not actively exercise. Demographic and socioeconomic details are shown in Table 1. Mean bowel movement increased from 2.04 ± 2.25 to 4.90 ± 2.70 in Group A and from 2.19 ± 2.06 to 5.30 ± 2.30 in Group B.

**Table 1:** Demographic and socioeconomic details

Parameters	Group A (sterilized probiotic with *L. helveticus* and 5.85 g polydextrose)	Group B (sterilized probiotic with *L. helveticus* without polydextrose)
Total number of subjects (*n*)	79	84
Gender, *n* (%)		
Male	17 (21.5%)	18 (21.4%)
Female	62 (78.5%)	66 (78.6%)
Median age (IQR), years	34 (26–37)	27 (22–35)
Ethnicity, *n* (%)		
Malay	58 (73.0%)	61 (73.0%)
Chinese	17 (22.0%)	23 (27.0%)
Indians	2 (2.5%)	0 (0.0%)
Others	2 (2.5%)	0 (0.0%)
Alcohol intake, *n* (%)		
Yes	1 (1.0%)	0 (0.0%)
No	78 (99.0%)	84 (100.0%)
Smoker, *n* (%)		
Yes	5 (6.0%)	1 (1.0%)
No	74 (94.0%)	83 (99.0%)
Fiber intake, *n* (%)		
Low	49 (62.0%)	52 (62.0%)
Medium	27 (34.0%)	30 (36.0%)
High	3 (4.0%)	2 (2.0%)
Physical exercise, *n* (%)		
Never	30 (38.0%)	25 (30.0%)
Sometimes	40 (51.0%)	48 (57.0%)
Habitually	9 (11.0%)	11 (13.0%)

*IQR* inter-quartile range

### Fecal pH

**Fig. 2:**
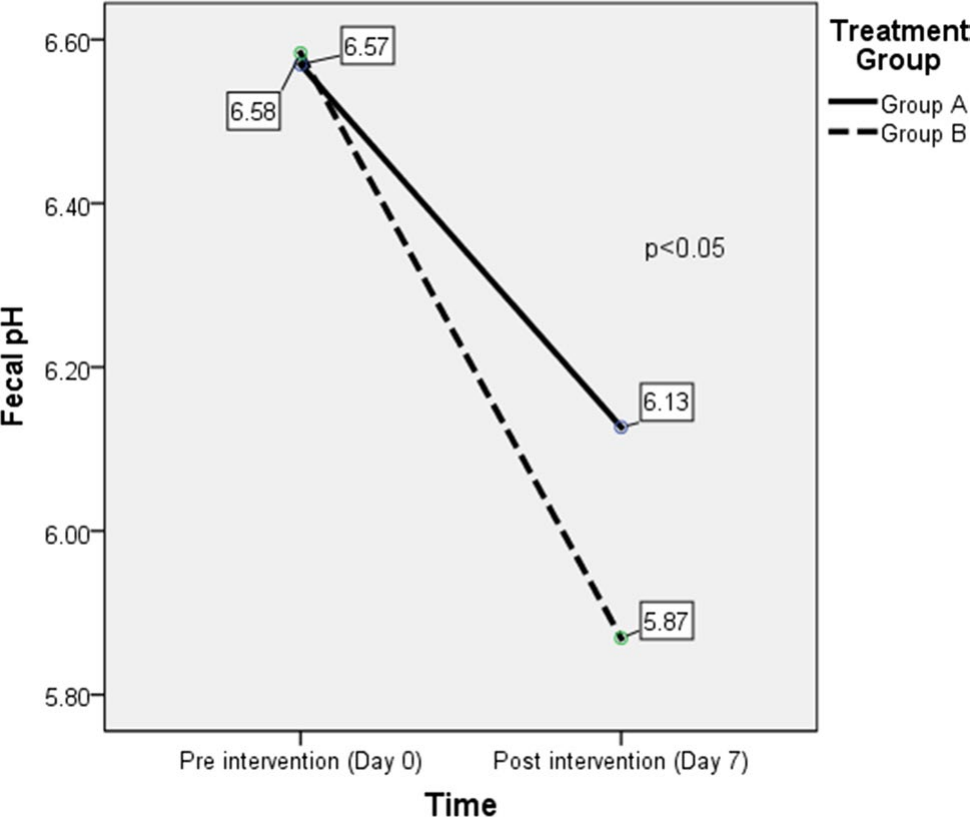
Mean comparisons of endpoint measures (fecal pH) between Group A (consumed sterilized probiotic with *L. helveticus* with 5.85 g polydextrose) versus Group B (consumed sterilized probiotic with *L. helveticus* without polydextrose)

The mean baseline fecal pH was nearly equal in both groups. Consumption of symbiotic and probiotic reduced fecal pH significantly. Fecal pH was normally distributed because of its skewness and kurtosis within ± 1. Median and mean value were similar. Most of the dots were on the line based on Q–Q Plot. Fecal pH for Groups A and B was reduced from 6.57 ± 0.96 to 6.13 ± 0.95 (*p *= 0.003) and 6.58 ± 1.0 to 5.87 ± 0.83 (*p *< 0.001), respectively. However, there was no statistical significant difference when compared between the two groups (*p *= 0.179) (Fig. 2).

### Intestinal Transit Time (ITT)

Subjects in both groups at baseline had longer ITT with marked variations between individuals (33–117 h) [29]. Post-intervention showed a significantly shorter transit time for both groups. The ITT was significantly reduced with median of 58 h (IQR = 43–72) reduced to 45 h (IQR = 24–59) and median of 48 h (IQR = 31–72) reduced to 30 h (IQR = 24–49) for Groups A and B, respectively (*p *< 0.01). However, there was no statistical significant difference between the two groups (*p *= 0.262). The results are summarized in Table 2.

**Table 2:** Median comparisons of endpoint measures (intestinal transit time) between Group A (sterilized probiotic containing *L. helveticus* and added 5.85 g polydextrose) versus Group B (sterilized probiotic containing *L. helveticus* without polydextrose)

Parameter	Study groups	Pre-intervention, day 0	Post-intervention, day 7	Intra-group *p* value
ITT (hours)	A	58 (43–72)	45 (24–59)	< 0.01
	B	48 (31–72)	30 (24–49)	< 0.01
Inter-group *p* value		0.075	0.262	

Data were expressed as median (IQR)

*IQR* inter-quartile range

### Fecal Weight

**Fig. 3:**
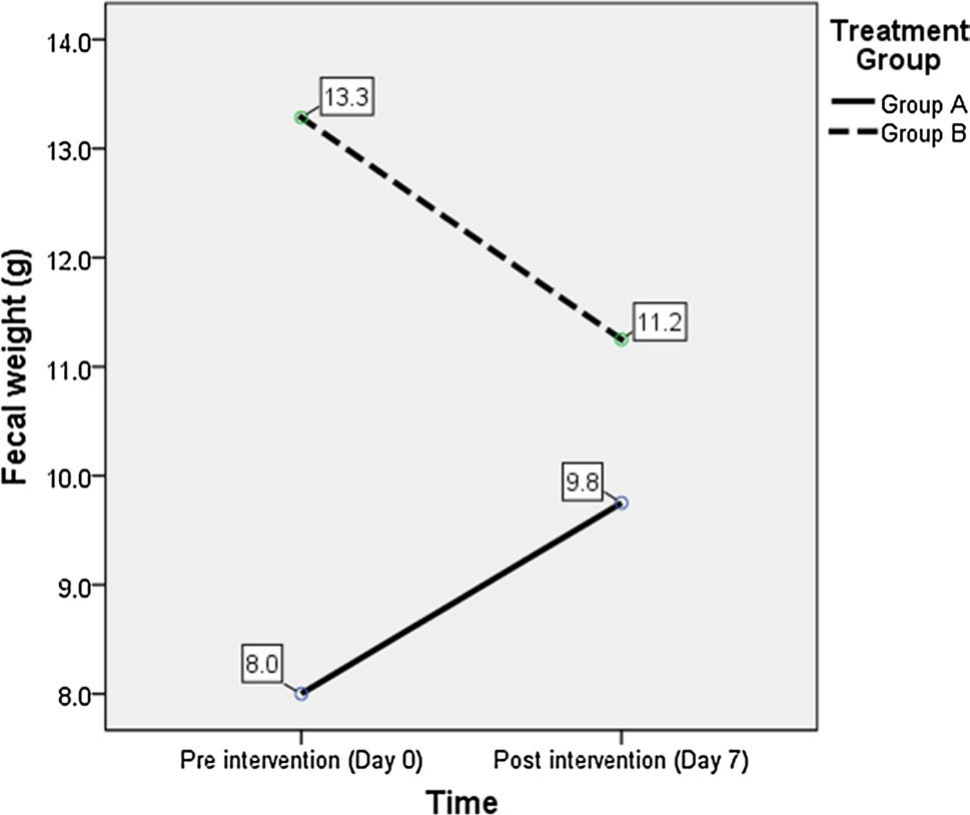
Mean comparisons of endpoint measures (fecal weight) between Group A (consumed sterilized probiotic with *L. helveticus* with 5.85 g polydextrose) versus Group B (consumed sterilized probiotic with *L. helveticus* without polydextrose)

The baseline mean for fecal weight was 8.0 ± 6.4 g in Group A and 13.3 g ± 19.4 g in Group B. After a week consumption of symbiotic, the mean fecal weight increased to 9.8 g ± 7.6 g (*p *= 0.003) in Group A. However, mean fecal weight reduced to 11.2 g ± 6.6 g (*p *= 0.308) in Group B. Fecal weight was normally distributed because of its skewness and kurtosis within ± 1. Median and mean value were similar. Most of the dots were on the line based on Q–Q plot. The results were not statistically significant between the two groups (*p *= 0.076) (Fig. 3).

### Constipation-Related Symptoms Assessment

**Fig. 4:**
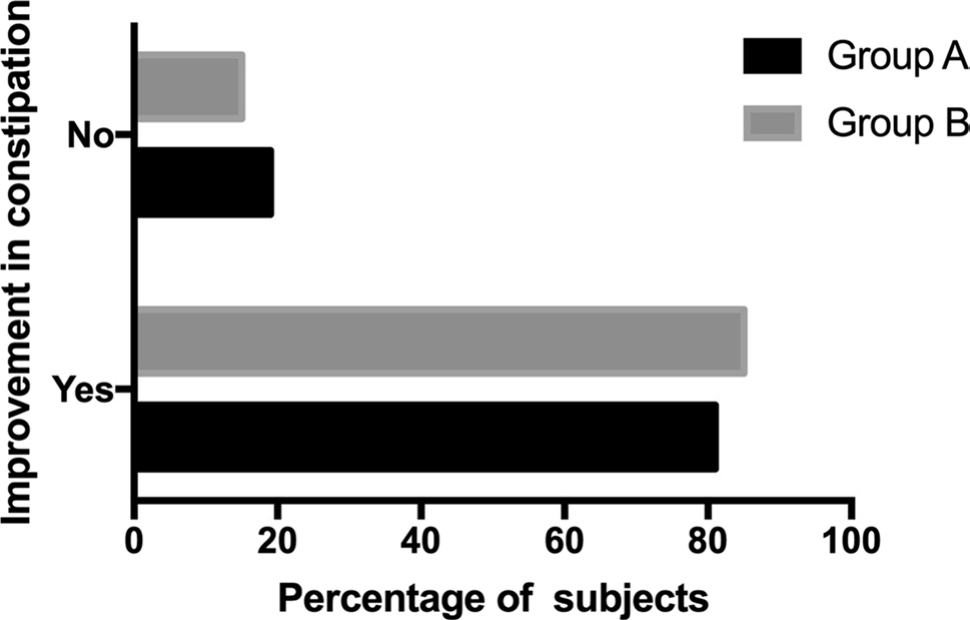
Mean comparisons of endpoint measures (improvement in constipation) between Group A (consumed sterilized probiotic with *L. helveticus* with 5.85 g polydextrose) versus Group B (consumed sterilized probiotic with *L. helveticus* without polydextrose)

**Fig. 5:**
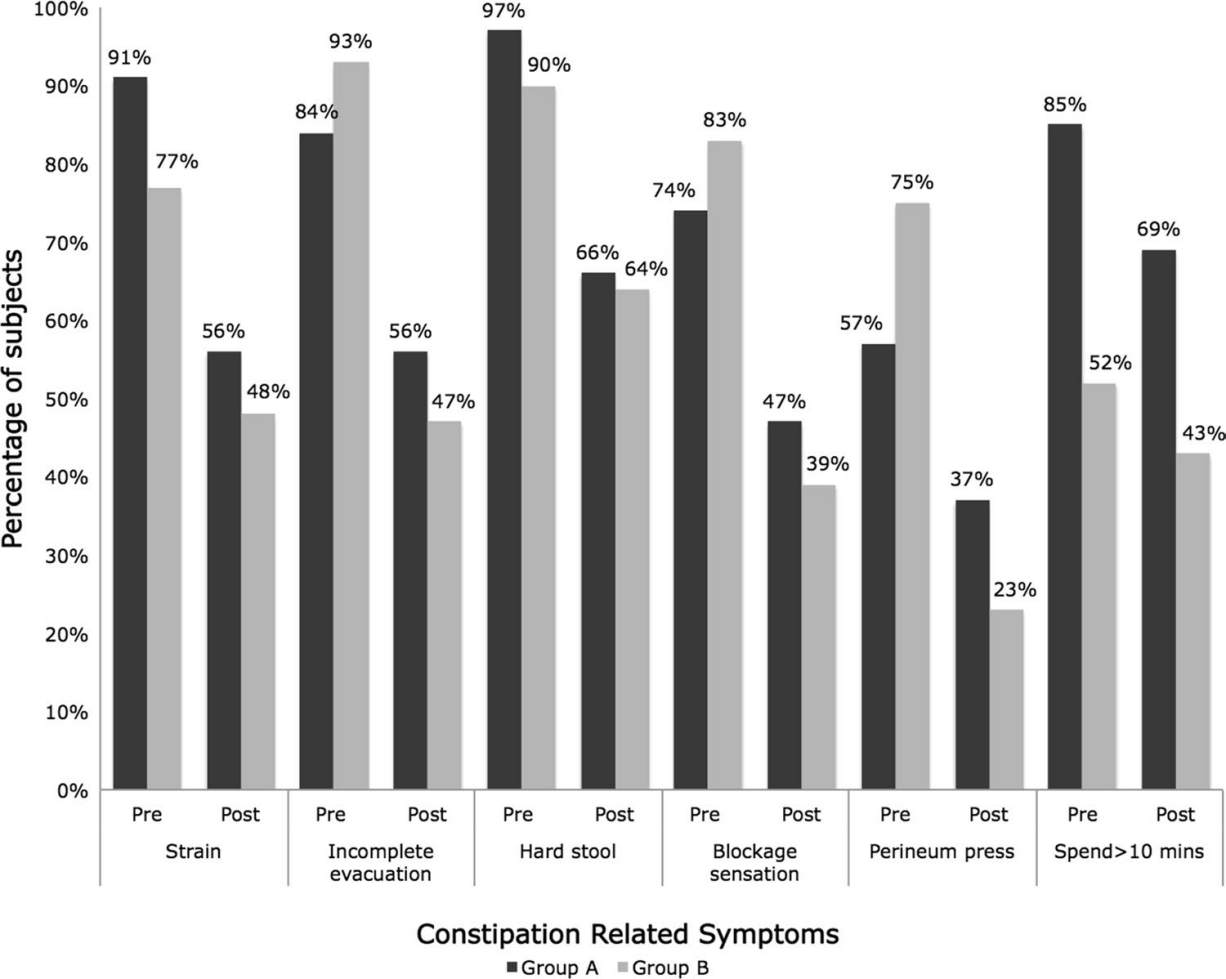
Mean comparisons of endpoint measures (constipation-related symptoms) between Group A (consumed sterilized probiotic with *L. helveticus* with 5.85 g polydextrose) versus Group B (consumed sterilized probiotic with *L. helveticus* without polydextrose)

Clinical responses were assessed using the modified *Garrigues* constipation questionnaires [28]. IBS-C-related symptoms for Groups A and B improved at the end of the study, and no major adverse event was reported in both groups. Of particular interest were the changes in constipation, incomplete evacuation, passing on hard stool, and spending more than 10 min to evacuate, and straining symptoms were reported with the highest frequency at baseline. For each of these symptoms, the relative decrease in symptom frequency was approximately twofold greater in the post-intervention group compared to baseline. Pre-intervention, all subjects in Groups A and B have constipation. Post-intervention, 81% of subjects in Group A significantly improved constipation, while in Group B, 84% claimed that they have no constipation (*p *< 0.05). In terms of straining during defecation, there was reduction from 91 to 56% versus 77 to 48% in Groups A and B, respectively. For incomplete evacuation, there was reduction from 84 to 56% versus 93 to 47% in Groups A and B, respectively. Similar improvements were observed for passing on hard stool and the need of spending more than 10 min to defecate. There was a reduction from 97 to 66% versus 90 to 64% in Groups A and B, respectively, in terms of having hard stool. Post-intervention showed a reduction in number of subjects spending more than 10 min for complete defecation (85–69% in Group A and 52–43% in Group B). Similar improvements were observed for other constipation-related symptoms post-intervention including fewer subjects having anal blockage sensation or the need of pressing perineum to defecate at the end of the study. Results are shown in Figs. 4 and 5. However, there was no statistical significance between these two groups (*p *= 0.67).

### Safety Evaluation

All subjects were compliant to the study protocol. Adverse events were mild with the most frequent adverse event being loose stool, reported in 22 of the subjects (27.8%) in Group A and 18 subjects (21.4%) in Group B, which may be seen as a beneficial effect. Another reported adverse event was mild abdominal discomfort, reported in one subject (1.3%) in Group A and two subjects (2.8%) in Group B. The abdominal discomfort did not require any medical intervention or hospitalization.

## Discussion

Constipation-predominant irritable bowel syndrome (IBS-C) is a common disorder in Malaysia with prevalence of 10.9–15.8% [4–6]. The variations in prevalence rates may be explained by the differences of the socioeconomic backgrounds of studied population. This current study was performed in an urban, the central part of Malaysia with the majority being Malays and Chinese population. Young Malay women were predominant in this study. This is consistent with Lovell et al. that reported prevalence of IBS appeared to be modestly higher in women, and they were prone to exhibit the constipation-predominant subtype as compared to men [30].

There are several evidences that indicated the changes in gut microbiota are fundamental in the pathogenesis and pathophysiology of IBS [31]. The microbiome may contribute to IBS symptoms by altering gut neuromotor-sensory function and the barrier function or influencing the gut–brain axis. Hence, numerous studies have reported the benefit of probiotics (cultured milk drink with *Lactobacillus* sp.) or prebiotics (polydextrose) in treating patients with IBS-C. However, data supporting the use of symbiotic remain sparse and whether symbiotic is superior to probiotic remains unclear. To the best of our knowledge, this is the first study in Malaysia comparing the effect of symbiotic versus probiotic among IBS-C patients.

European Food Safety Authority Panel in 2010 stated that consumption of fiber-containing foods greater than 25 g per day will give great benefits in terms of improving health well-being, reducing the risk of coronary heart disease and type 2 diabetes, and even helping in weight maintenance among adults [32]. Fiber intake of 25 g per day for women and 38 g per day for men are currently recommended among adults in Malaysia based on the 2017 Recommended Nutrient Intake of Adult [33]. However, it is known that the majority of Malaysian adults have reduced fiber intake in their diets [34].

Incorrect diet may explain gastrointestinal motility disorders or dyssynergic defecation [9]. Fiber may improve bowel habits if it is taken together with adequate fluids. It stimulates the growth of colonic flora and hence increases fecal mass. There is a dose–response correlation between the amount of fiber intake, water intake, and the amount of fecal output [35]. Despite the benefits of fibers, it is important to be aware that fiber overdosed may paradoxically increase risk of bloating and abdominal distension. This may lead to poor adherence of regular fiber consumption [36].

In our study, *L. helveticus* species were prepared in sterilized probiotic with or without polydextrose. Fecal pH for both groups was reduced significantly after only 1 week of consumption of the treatment drinks. Fecal pH among patients with IBS-C is higher than normal populations, and microorganisms such as *Bifidobacteria* may lower colonic pH by producing lactic acid, acetic acid, and other acids [37]. Besides that, Jie et al. [18] had studied the physiological effect of polydextrose intake among Chinese and reported that there was a reduction in the bowel pH after consumption. Substantial changes in fecal anaerobes were also seen after polydextrose intake. *Lactobacillus* and *Bifidobacterium* species were increased, while bacteroides species (*B. fragilis, B. vulgatus*, and *B. intermedius*) were decreased. These changes in the gut microbiota were responsible for the reduction in fecal pH [18]. However, there was no significant difference between the two groups. The short duration of treatment may be a contributing factor to the nonsignificant difference found between the two groups.

Baseline ITT was more than 48 hours in both treatment groups. There was a significant reduction in the median ITT by 13 h versus 18 h in Groups A and B, respectively. Our subjects reported increased bowel opening with reduction of ITT. Subjects with near normal ITT did not suffer from expected diarrhea. Low intake of fiber is another associated factor for IBS-C. Fiber can stimulate the growth of colonic flora, thereby increasing fecal mass. In 2005, Rees et al. [21] reported that fiber increases fecal mass after 8–12 weeks of intervention as compared to placebo. We have observed an increment of mean fecal weight in the symbiotic group. However, the increment was not statistically significant in comparison with Group B. The method to determine fecal weight has been argued by previous researchers as it is imprecise to be useful. Therefore, we did not discuss further on this result as it has a lack of standardization on the amount of feces sent for analysis.

Guyonnet et al. showed that an improvement in gastrointestinal function in IBS-C subjects was observed after the usage of *B. lactis* for 6 weeks [38]. However, with only 1-week intervention in our study, both treatment groups showed sustained significant improvement in all constipation-related symptoms and bowel opening increased at the end of treatment, which suggested a more immediate action mechanism. It is not known whether this response was purely due to the probiotic effect and synbiotic effects or perhaps due to the variation in day-to-day diet.

Many chronic constipation studies apply intervention period of between 8 and 12 weeks, which is in line with recommendations for trial design in functional gut disorders [39, 40]. Modulation of the human intestinal microflora by consumption of probiotic or symbiotic may require a certain amount of time to translate the changes into improvement of bowel movement. Surprisingly, in our study, significant improvements were seen in the reduction of fecal pH, ITT, and improvement of constipation-related symptoms even after only a week of intervention period in both groups. Theoretically, synbiotic administration may improve the survival of probiotic and restore intestinal microbial balance, which should confer a better positive effect on the treatment of IBS-C. However, there was no statistical significant difference found between the two groups, possibly due to the short intervention period. Further investigation with a longer duration period of eight to 12 weeks is warranted in order to obtain conclusive and perhaps significant results regarding the superiority of symbiotic over probiotic.

One of the limitations identified in our study is the lack of placebo test group using probiotic without live cultures and polydextrose. This placebo group is not included in the study as we were focused on investigating the beneficial effects of polydextrose in sterilized probiotic containing *L. helveticus* (synbiotic) on improving IBS-C subjects’ conditions. Due to this, the placebo effects and influences of probiotic without live cultures on the condition are unknown. Furthermore, despite sterilized probiotic being used for both groups, it was observed that there was a significant decrease in ITT in both groups. There are some possibilities that other materials, such as the presence of lactic acid in the sterilized probiotic, that may have caused this effect. However, we should consider a limitation in assessing ITT whereby ITT assessment was solely based on response from a subject’s observation on change of stool color. Another limitation in this study was regarding the relationship of daily dietary intake during study and its correlation with improvement of IBS symptoms observed in the majority of the subjects post-intervention. Studies have shown diet plays a major role in modulating gastrointestinal microbiota especially among IBS-C subjects. It is suggested that probiotic is able to improve symptoms of constipation in IBS, even though it does not contain any living cultures. However, more studies should be conducted in the future for further validations.

## Conclusion

In conclusion, a combination of dietary fibers in the form of polydextrose and probiotic did not appear to confer better health benefits in subjects with irritable bowel syndrome than probiotics alone. However, daily consumption of probiotic containing *L. helveticus* for a week improved constipation-related symptoms, reduced fecal pH, and shortened ITT among subjects with IBS-C. In future, longer duration of study would be helpful to ascertain the non-superiority of symbiotics compared to probiotics alone.
